# Supplementary material for: A compressed hierarchy for visual form processing in the tree shrew
Source: Nature. 2025 Aug 27;646(8086):872–82. doi: 10.1038/s41586-025-09441-w (PMC12545169; doi:10.1038/s41586-025-09441-w)
Supplement: Supplementary file 1 — Reporting Summary [file 41586_2025_9441_MOESM1_ESM.pdf]

nature portfolio

Corresponding author(s): Frank F. Lanfranchi, Doris Y. Tsao

Last updated by author(s): Apr 21, 2025

Reporting Summary

Nature Portfolio wishes to improve the reproducibility of the work that we publish. This form provides structure for consistency and transparency in reporting. For further information on Nature Portfolio policies, see our Editorial Policies and the Editorial Policy Checklist.

Statistics

For all statistical analyses, confirm that the following items are present in the figure legend, table legend, main text, or Methods section.

n/a	Confirmed	
☐	☒	The exact sample size (*n*) for each experimental group/condition, given as a discrete number and unit of measurement
☐	☒	A statement on whether measurements were taken from distinct samples or whether the same sample was measured repeatedly
☐	☒	The statistical test(s) used AND whether they are one- or two-sided<br>*Only common tests should be described solely by name; describe more complex techniques in the Methods section.*
☒	☐	A description of all covariates tested
☐	☒	A description of any assumptions or corrections, such as tests of normality and adjustment for multiple comparisons
☐	☒	A full description of the statistical parameters including central tendency (e.g. means) or other basic estimates (e.g. regression coefficient) AND variation (e.g. standard deviation) or associated estimates of uncertainty (e.g. confidence intervals)
☐	☒	For null hypothesis testing, the test statistic (e.g. *F*, *t*, *r*) with confidence intervals, effect sizes, degrees of freedom and *P* value noted<br>*Give P values as exact values whenever suitable.*
☒	☐	For Bayesian analysis, information on the choice of priors and Markov chain Monte Carlo settings
☒	☐	For hierarchical and complex designs, identification of the appropriate level for tests and full reporting of outcomes
☐	☒	Estimates of effect sizes (e.g. Cohen's *d*, Pearson's *r*), indicating how they were calculated

Our web collection on statistics for biologists contains articles on many of the points above.

Software and code

Policy information about availability of computer code

Data collection	We listed all softwares used in the experiments and for analysis in the Methods section.<br>- OpenEphys for electrophysiology<br>- EyeLink system for eye tracking.
Data analysis	We listed all softwares used in the experiments and for analysis in the Methods section.<br>- Electrophysiological data was spike sorted using Kilosort (version 2.5 and 3) and manually curated in Phy(version2, https://github.com/cortex-lab/phy?tab=readme-ov-file).<br>- AlexNet (https://github.com/BVLC/caffe/tree/master/models/bvlc_alexnet original version from Krizhevsky A, Sutskever I, Hinton GE. "ImageNet Classification with Deep Convolutional Neural Networks." Advances in neural information processing systems. 2012.)<br>Custom code written in Python was used for analysis, which is available from the lead corresponding author upon reasonable request.

For manuscripts utilizing custom algorithms or software that are central to the research but not yet described in published literature, software must be made available to editors and reviewers. We strongly encourage code deposition in a community repository (e.g. GitHub). See the Nature Portfolio guidelines for submitting code & software for further information.

Data

Policy information about availability of data

All manuscripts must include a data availability statement. This statement should provide the following information, where applicable:

- Accession codes, unique identifiers, or web links for publicly available datasets
- A description of any restrictions on data availability
- For clinical datasets or third party data, please ensure that the statement adheres to our policy

Data and code supporting the findings of this paper are available on request from the corresponding authors.

Research involving human participants, their data, or biological material

Policy information about studies with human participants or human data. See also policy information about sex, gender (identity/presentation), and sexual orientation and race, ethnicity and racism.

Reporting on sex and gender	N/A
Reporting on race, ethnicity, or other socially relevant groupings	N/A
Population characteristics	N/A
Recruitment	N/A
Ethics oversight	N/A

Note that full information on the approval of the study protocol must also be provided in the manuscript.

Field-specific reporting

Please select the one below that is the best fit for your research. If you are not sure, read the appropriate sections before making your selection.

☒ Life sciences ☐ Behavioural & social sciences ☐ Ecological, evolutionary & environmental sciences

For a reference copy of the document with all sections, see nature.com/documents/nr-reporting-summary-flat.pdf

Life sciences study design

All studies must disclose on these points even when the disclosure is negative.

Sample size	No statistical methods were used to pre-determine sample sizes, but our sample sizes were similar to those used in previous publications in both tree shrews such as Lee KS, Huang X, Fitzpatrick D. "Topology of ON and OFF inputs in visual cortex enables an invariant columnar architecture." Nature (2016) and macaques such as Bao P, She L, McGill M, Tsao DY. "A map of object space in primate inferotemporal cortex." Nature (2020).
Data exclusions	No datasets were excluded from analysis but some exclusion criteria for single neurons were applied when appropriate (e.g. when a neuron was not visually responsive to the visual stimulus presented, it does not make sense to include it in the further steps of the analysis). A full and detailed section is included in the Method section under ' Visually responsive cells'.
Replication	Results were replicated across at least 2-3 different animals for each experiment independently.
Randomization	This is not applicable for this study because of no data grouping.
Blinding	This is not applicable for this study because of no data grouping.

Reporting for specific materials, systems and methods

We require information from authors about some types of materials, experimental systems and methods used in many studies. Here, indicate whether each material, system or method listed is relevant to your study. If you are not sure if a list item applies to your research, read the appropriate section before selecting a response.

Materials & experimental systems

n/a	Involved in the study
☒	☐ Antibodies
☒	☐ Eukaryotic cell lines
☒	☐ Palaeontology and archaeology
☐	☒ Animals and other organisms
☒	☐ Clinical data
☒	☐ Dual use research of concern
☒	☐ Plants

Methods

n/a	Involved in the study
☒	☐ ChIP-seq
☒	☐ Flow cytometry
☒	☐ MRI-based neuroimaging

Animals and other research organisms

Policy information about studies involving animals; ARRIVE guidelines recommended for reporting animal research, and Sex and Gender in Research

Laboratory animals	Five tree shrews (Tupaia Belangeri) aged 6 moths to 2.5 years old and three male rhesus macaques (Macaca mulatta) aged 5-15 years old were used in this study.
Wild animals	The study did not involve wild animals.
Reporting on sex	This study was conducted using male and female tree shrews. Only male macaques were used.
Field-collected samples	The study did not involve field-collected samples.
Ethics oversight	All procedures conformed to local and US National Institutes of Health guidelines, including the US National Institutes of Health Guide for Care and Use of Laboratory Animals. All experiments were performed with the approval of the Caltech and UC Berkeley Institutional Animal Care and Use Committee.

Note that full information on the approval of the study protocol must also be provided in the manuscript.

Plants

Seed stocks	N/A
Novel plant genotypes	N/A
Authentication	N/A
